# Next‐Generation Sequencing Methods for Sensitive Hepatitis B Viral Genome Analysis: A European Study

**DOI:** 10.1002/jmv.71130

**Published:** 2026-09-02

**Authors:** Michael X. Fu, Maria F. Perdomo, Sheila F. Lumley, Johan Ringlander, Kai Kean, Kaitlin Reid, Richard Mayne, Oscar Enrique Torres Montaguth, Leysa Forrest, Sarah Buddle, Johannes C. Botha, Joakim B. Stenbäck, Zachery Dickson, Chris Kent, Haiting Chai, Matthew Byott, Leo Hannolainen, Shannah Secret, George Airey, Klaus Hedman, Monique I. Andersson, M. Azim Ansari, Eleni Nastouli, Judith Breuer, Philippa C. Matthews, Tanya Golubchik, William L. Irving, Peter Simmonds, Heli Harvala

**Affiliations:** ^1^ Nuffield Department of Medicine University of Oxford Oxford UK; ^2^ Department of Virology University of Helsinki Helsinki Finland; ^3^ Department of Infectious Diseases and Microbiology Oxford University Hospitals NHS Foundation Trust Oxford UK; ^4^ Department of Clinical Microbiology Sahlgrenska University Hospital Gothenburg Sweden; ^5^ Department of Infectious Diseases Institute of Biomedicine, University of Gothenburg Gothenburg Sweden; ^6^ Infection, Immunity and Inflammation Department, Great Ormond Street Institute of Child Health University College London London UK; ^7^ Genetics and Genomic Medicine Department, Great Ormond Street Institute of Child Health University College London London UK; ^8^ Advanced Pathogen Diagnostics Unit University College London Hospitals NHS Trust London UK; ^9^ Institute of Microbiology and Infection University of Birmingham Birmingham UK; ^10^ Radcliffe Department of Medicine University of Oxford Oxford UK; ^11^ Division of Infection and Immunity University College London London UK; ^12^ The Francis Crick Institute London UK; ^13^ Sydney Infectious Diseases Institute, School of Medical Sciences, Faculty of Medicine and Health University of Sydney Sydney Australia; ^14^ NIHR Nottingham Biomedical Research Centre Nottingham University Hospitals NHS Trust and the University of Nottingham Nottingham UK; ^15^ Institute of Biomedicine University of Turku and University Hospital of Turku Turku Finland; ^16^ Microbiology Services, NHS Blood and Transplant Colindale UK

**Keywords:** clinical microbiology, diagnostics, high‐throughput sequencing, Illumina, nanopore, sensitivity, viral metagenomics, virus detection

## Abstract

This multicentre study investigated the utility of next‐generation sequencing (NGS) to detect and generate hepatitis B virus (HBV) genomes in samples of low viral load (from 0.2 to 6207 IU/mL). 23 HBV DNA‐positive plasma samples of genotypes A‐E and one HBV‐negative control sample were assayed blindly via 9 established NGS methods from 6 European laboratories. Methods included untargeted metagenomics, pre‐enrichment by probe‐capture followed by Illumina sequencing, and HBV‐specific PCR pre‐amplification followed by sequencing with Nanopore or Illumina. Full HBV genomes were obtained only from samples with viral loads > 1000 IU/mL using probe‐capture methods, > 200 IU/mL using PCR‐Illumina methods, > 10 IU/mL using PCR‐Nanopore methods, and in no samples using metagenomic methods. Contamination was observed in the negative control and samples with very low viral loads in PCR‐based methods. Probe‐capture and metagenomic methods detected additional viruses not routinely screened in blood donations, including polyomaviruses and herpesviruses; positive results were confirmed by PCR. In conclusion, NGS may delineate whole‐genome sequences at low viral loads if supported by a PCR pre‐amplification step. Probe‐capture methods also reliably detect HBV without pre‐amplification but show limited genome coverage for samples with low viral loads; they may additionally detect a wide range of blood‐borne viruses.

## 1 Introduction

The advent of high‐throughput sequencing platforms in the mid‐2000s, coined ‘next‐generation sequencing’ (NGS), led to dramatic advances in the characterisation of host and pathogen genomes in health and disease [1]. Metagenomics, a commonly used NGS method to sequence all genomic material in a sample, has been instrumental in detecting broad‐spectrum microbes, surveillance of emerging infections, and identifying novel pathogens [2, 3]. The wider adoption of NGS in clinical microbiology and public health has been hindered by complex methodological workflows, complications in the detection and reporting of contaminating sequences, and a reduced sensitivity compared to real‐time and nested PCR assays [2, 4]. In particular, the inadequate sensitivity of untargeted metagenomics has constrained its application for genomic analysis of hepatitis B virus (HBV) in samples in the low viral load range often found in clinical samples; the limited representation of target HBV sequences in metagenomic libraries can make genomic characterisation challenging [5, 6].

HBV remains a global public health issue; the virus chronically infects around 246 million individuals worldwide, accounting for 1.1 million HBV‐related deaths in 2022 [7]. In the blood, HBV exists in relaxed‐circular DNA (rcDNA) form: a partially double‐stranded genome of approximately 3.2 kb, consisting of overlapping reading frames encoding four genes: polymerase, surface, precore/core, and X. HBV can be classified into 10 genotypes (A to J) based upon sequence divergence ≥ 8%, with inter‐genotypic differences in geographic distribution, transmission mode, and clinical outcomes [8]. Sequencing full‐length HBV genomes identifies (sub)genotypes and recombinants and provides a better understanding of mechanisms of transmission and persistence, identifies treatment‐resistant and immune‐escape mutations, and thus can aid in the development of new therapeutics [9]. Furthermore, whole‐genome sequencing (WGS) of low viral load (VL) samples enables the characterisation of occult HBV infection and the assessment of viral clearance, persistence and reactivation.

Methodologies used in clinical diagnostics for sequencing viral genomes need to be time‐ and cost‐efficient, specific, and sensitive to support their integration into clinical, public health, and research laboratories [10]. Previous evaluations of viral sequencing methods have been limited to individual workflows and laboratories, with few coordinated comparisons, typically using synthetic or low‐diversity materials due to limited clinical sample availability [11]. Method comparisons using clinical samples of varying loads are limited, but include a previous investigation of the effectiveness of NGS methods for the characterisation of hepatitis C virus detection frequencies and genome coverage [12]. The value of many studies is limited by the inclusion of samples with relatively high VLs without determining the detection limits of different NGS methods, for example [11].

Amid calls for the HBV field to work progressively toward developing NGS methods [8], this study brought together six European laboratories with nine established virus sequencing protocols, encompassing four commonly used NGS methods. First, the untargeted metagenomics method sequenced all genomic material in the samples. The second method involved the addition of biotinylated nucleic acid probes to enrich for large numbers of known pathogens and selectively capture target sequences before Illumina sequencing. The final methods involved PCR amplification of HBV fragments, followed by sequencing using the short‐read Illumina or the long‐read Oxford Nanopore Sequencing (ONT) platforms. All methods except untargeted metagenomics have previously demonstrated the ability to read and assemble whole HBV genomes from clinical samples [3, 6, 10, 13, 14].

The four NGS methods were evaluated using a panel of 23 HBV DNA‐positive plasma samples with low VLs. Through this comparison, we assessed the ability of NGS to detect and generate HBV sequences, as well as to co‐detect other viruses potentially present in the samples. Overall, this study aims to provide an overview of the HBV‐specific sensitivities of currently applied NGS methodologies and to evaluate their potential suitability for sequencing low VL samples in clinical practice.

## 2 Materials and Methods

### 2.1 Samples

Twenty‐three large‐volume samples, each containing around 200 mL of plasma, from individuals infected with HBV genotypes A, B, C, D, and E were obtained from blood donors from NHS Blood and Transplant (NHSBT). Typical of the HBV serological profiles observed in voluntary blood donor populations, 20 of 23 samples were reactive in two HBsAg assays (Murex and BioRad), whilst all 23 were total anti‐HBc positive (Murex, Diasorin), anti‐HBc IgM negative; one sample was HBeAg‐positive (VIDAS), and all 23 samples were anti‐HBe positive (VIDAS, Liaison XL). To reflect the heterogeneity in the extraction methods used by NGS protocols and account for the non‐negligible stochastic effect of measuring low VLs, geometric mean VLs were calculated across eight separate real‐time PCR measurements from eight extractions (five of which were obtained from a previous investigation [15] and three further extractions from 5 mL of plasma). The samples had geometric mean VLs ranging from 0.2 to 6207 IU/mL (approximately 1–31,000 copies/ml). Furthermore, a control plasma sample (around 200 mL) that screened negative for HBV serological markers and other blood‐borne infections was obtained from NHSBT, where HBV DNA negativity was independently confirmed by real‐time PCR measurements. All donors provided signed consent to use their samples for research purposes. This study was approved by the Blood Supply Clinical Audit, Risk and Effectiveness Committee of NHSBT (numbers 22031 and 23002). All 24 samples, including the negative control, were sent to each laboratory on dry ice, with anonymised sample identifiers and no other sample information. As plasma samples were used, sequencing predominantly reflected circulating virion‐associated HBV DNA (rcDNA). Intracellular forms, such as covalently closed circular DNA (cccDNA), were therefore not assessed. Although cccDNA has not been demonstrated in plasma, other intracellular‐derived forms, notably integrated HBV DNA, have been detected in plasma and may contribute to detected sequences, especially in samples with low VL [14, 16, 17].

### 2.2 Sequencing Methods

The panel of 24 blinded samples was used to evaluate the performance of nine established NGS protocols at six expert European laboratories, performed in the years 2023–2024. Methods included two untargeted metagenomic protocols using Illumina (MTG‐A and MTG‐B), three pan‐viral probe‐based enrichment protocols using Illumina (TAC‐A, TAC‐B, and TAC‐C), two HBV‐specific PCR amplification protocols using Illumina (PCR‐A and PCR‐B [PCR‐Illumina]) and two HBV‐specific PCR amplification protocols using ONT (PCR‐1 and PCR‐2 [PCR‐ONT]). The protocols were specific to each laboratory, and the impact of individual methodological variables could not be evaluated independently within this study. Further details of protocols are found in the supplementary material. Of note, the input plasma volumes for extractions ranged from 0.2 to 20 mL. Extracted nucleic acids underwent library preparation according to local protocols, where input volumes ranged from 5 to 50 μL. Effective test volumes ranged from 4 to 800 μL. The pre‐capture libraries for TAC‐B were sequenced for MTG‐B; thus, the samples were prepared similarly for both protocols.

Factors relating to the technical practicality and costs associated with the use of NGS assays greatly influence their adoption for clinical diagnostics. Laboratories were asked to provide the total time and cost of each protocol step from extraction to sequencing on a standardised template document, considering the typical number of samples that would normally be included per run for each protocol. Times for each stage were separated into per‐person labour time and waiting times (which included machine running times).

### 2.3 Bioinformatic Processing

Post‐read filtering (including deduplication of reads or removal of PCR duplicates) and genome assembly (either mapping to the closest available reference or de novo) were performed using established protocol‐specific pipelines at each centre (see supplementary material). Sequences were standardised to begin at the EcoR1 restriction site (the conventional origin of the genome [18]). CSV files for each assembled genome/sample were then sent from each centre, containing the number of base calls at each nucleotide site. These files were received at the coordinating laboratory, where further bioinformatics analyses were performed using a common set of tools on Microsoft Excel (version 16.103) and SSE [19, 20] (version 1.4) as previously described [12]. For each individual assembled genome, a 95% majority base consensus sequence was calculated at each genome position possessing one or more base reads, incorporating the calling of ambiguous bases when necessary. Assembled genome sequences were examined, and data on base frequencies at each site were realigned to ensure consistent numbering. These were also used for downstream analyses of base counts and calculation of genome coverage and Shannon entropy.

Within‐sample consensus sequences were assembled for each sample from the different NGS protocols by combining more than one similar assembled sequence with genome coverage of around 100% using the programme Sequence Join in the SSE package [20], followed by visual inspection of sites with ambiguous bases. Specific sequences from each method were compared with the within‐sample consensus, and the numbers of nucleotide differences were recorded using the programme Sequence Dist [20]. Insertions and deletions were included in consensus generation and sequence comparisons where consistently detected across protocols. Sites with missing bases or with insertions and deletions not detected by one of the protocols being compared were not included in that pairwise comparison of sequence identity. Thresholds for base‐calls were set to practically represent the realities of sequencing: a threshold of one base read per nucleotide site was utilised for MTG and TAC methods, whilst a more stringent minimum threshold of 10 base reads at each nucleotide site was applied for PCR‐based methods.

Within‐sample consensus NGS sequences were (sub)genotyped as previously described [15]. When whole‐genome assemblies could not be constructed (i.e. no two complete NGS sequences were similar), genotypes were defined from either the most complete NGS sequence or Sanger sequence, the latter if there were no similar NGS sequences. In these cases, individual assembled NGS sequences were compared relative to other available NGS and Sanger sequences, when possible. Individual NGS sequences from each protocol were genotyped using the Genome Detective system [21].

### 2.4 Nested PCR and Sanger Sequencing

Sequences of a portion of the surface gene obtained previously from extensive nested PCR and Sanger sequencing were included for comparison to the NGS sequences [22] (see Figure S1 for phylogenetic tree of all HBV‐positive samples obtained via Sanger sequencing of nested PCR amplicons). Using DNA extracted from 5 mL of plasma [15], nested PCR and sequencing of two further regions were performed: one spanning the surface and polymerase genes, and another spanning the X and precore/core genes (see Table S2 for primer details and nucleotide positions).

### 2.5 Definitions

Certain terms used in this manuscript have been defined to aid readability (see supplementary material).

### 2.6 Detection of Other Viruses by PCR

Since HBV was not the only possible virus sequenced from the five TAC and MTG protocols from three laboratories, the deduplicated read counts and genome coverage percentages were obtained for any additional viruses detected from the sample panel, following the full bioinformatics pipelines for each centre. When a certain virus was detected by more than one NGS protocol in the same sample, confirmatory PCR was performed in all samples (see supplementary material).

### 2.7 Statistical Analyses

Spearman's correlation was used to assess associations between VL and sequencing metrics across nine protocols. *P*‐values were adjusted for multiple testing within each set of comparisons using the Benjamini‐Hochberg method. *α* was 0.05. Data analyses and visualisation were performed with GraphPad Prism (v10.4.2, LLC), except *p*‐value adjustment performed with Microsoft Excel (version 16.103).

## 3 Results

### 3.1 HBV Detection and Accuracy of Assembled Sequences

HBV amplicons were obtained for all 23 samples by Sanger sequencing. Within‐sample consensus NGS sequences were constructed for 14/15 samples with VL above 50 IU/mL (Table 1). Sequences assembled using different NGS protocols were generally identical or nearly identical to the within‐sample consensus. A few individual sequences were > 5% divergent from the within‐sample consensus (shown in pink), indicating a different contaminating HBV strain. Assembled sequences were compared for eight samples with lower VLs, for which within‐sample consensus sequences were unavailable. Different HBV genotypes were detected by at least one protocol for all eight samples (Table S3). Three samples, VLs 1.1, 1.8, and 8.5 IU/mL, yielded discrepant sequences in all NGS protocols.

**Table 1:** Overview of the detection of HBV in sample sets.

		PCR (Nanopore)		PCR (Illumina)		Target capture			Metagenomics				Sanger sequencing		
Sample	Viral load (IU/mL)	**PCR‐1**	**PCR‐2** ^**1**^	**PCR‐A**	**PCR‐B** ^**1**^	**TAC‐A** ^**2**^	**TAC‐B** ^**2**^	**TAC‐C**	**MTG‐A**	**MTG‐B** ^**2**^	**Within‐sample consensus**	**Genotype assigned**	**S**	**SP**	**XC**
HBV15	6207.0	100%	100%	91%	100%	11%	100%	90%	25%	72%	**Y**	**A2**	12%	30%	22%
HBV8	3154.0	100%	99%	98%	100%	91%^c^	97%	99%	50%	14%	**Y**	**D1**	12%	NS	NS
HBV2	3108.0	100%	90%	NS	100%	90%^c^	81%	100%	NS	25%	**Y**	**A2**	12%	NS	22%
HBV7	1058.0	100%	100%	98%	99%	17%	83%	93%	11%	25%	**Y**	**C1**	12%	29%	22%
HBV19	859.8	100%	49%	NS	100%	22%	4%	88%	10%	NS	**Y**	**D3**	12%	31%	22%
HBV16	599.2	100%	85%	59%	100%	9%	NS	75%	NS	NS	**Y**	**A1**	12%	NS	NS
HBV1	551.6	100%	50%	76%	100%	6%	38%	88%	4%	NS	**Y**	**D2**	12%	NS	NS
HBV14	522.3	100%	85%	92%	100%	9%	95%	80%	NS	49%	**Y**	**E**	12%	30%	NS
HBV23	337.1	100%	57%	99%	98%	64%	71%	93%	NS	16%	**Y**	**D4**	12%	31%	22%
HBV17	299.9	100%	100%	77%	60%	NS	14%	50%	NS	NS	**Y**	**E**	12%	30%	NS
HBV9	138.1	100%	100%	33%	99%	9%	8%	35%	2%	NS	**Y**	**A1**	12%	30%	NS
HBV5	128.4	100%	NS	NS	57%	NS	NS	66%	NS	NS	**N**	**E**	12%	30%	N
HBV22	73.6	100%	54%	NS	77%	NS	NS	54%	NS	NS	**Y**	**D2**	12%	31%	NS
HBV4	60.5	100%	85%	59%	100%	35%	7%	100%	12%	NS	**Y**	**B4**	12%	30%	22%
HBV13	60.4	100%	85%	NS	94%	NS	NS	91%	5%	NS	**Y**	**D1**	12%	31%	22%
HBV21	41.0	100%	37%	NS	73%^a^	NS	7%	43%	NS	NS	**N**	**A**	12%	30%	NS
HBV6	11.3	100%	NS	NS	57%	NS	3%	28%	NS	3%	**N**	**D3**	12%	31%	NS
HBV18	8.5	NS	37%	NS	33%	NS	NS	NS	NS	NS	**N**	**A**	12%	NS	NS
HBV20	3.3	100%	17%	NS	43%^a^	NS	NS	26%	< 1%	NS	**N**	**D**	12%	NS	NS
HBV12	1.8	NS	17%	NS	56%^a^	NS	NS	25%	NS	NS	**N**	**A**	12%	NS	NS
HBV24	1.1	NS	NS	NS	71%^b^	NS	NS	NS	NS	NS	**N**	**E**	12%	NS	NS
HBV11	0.3	99%	4%	NS	29%^b^	NS	NS	NS	NS	NS	**N**	**D**	12%	NS	NS
HBV3	0.2	NS	52%	NS	74%^a^	NS	NS	5%	NS	NS	**N**	**A3**	5%	NS	NS
Negative (HBV10)	—	100%^#^	17%	48%	45%	NS	NS	NS	NS	NS	**N**	**—**	NS	NS	NS

Within‐sample consensus	Shading key					
**Y (% sequence identity to within‐sample consensus)**						
	No sequence	≥ 85%	≥ 90%	≥ 95%	≥ 98%	≥ 99.5%
**N (similarity of sequences)**						
	No sequence	Different sequence to others	Similar sequence			

*Note:* The percentage genome completeness is given in each cell. The left‐hand side of the table with blue headers shows the sequences obtained from the NGS methods, whilst the right‐hand side (calamine colour) shows the sequences obtained by Sanger sequencing (length of S amplicon = 395 nucleotides; SP = 973 nucleotides; XC = 698 nucleotides). Within‐sample consensus sequences were assembled for each sample from the individual sequences of the NGS methods that had around 100% genome coverage and were genetically similar, which were assigned to HBV genotypes. The percentage genetic relatedness of the sequences from each method was compared to the within‐sample consensus sequence, where assembled sequences similar to the within‐sample consensus were shaded on a grayscale, and different strains (differing in > 5% nucleotide sequence from the within‐sample consensus) were shaded in a pink scale. The absence of sequences was noted by NS. Where within‐sample consensus sequences could not be assembled (i.e. no two complete sequences were similar), genotypes were defined from Sanger sequences, and the NGS sequences were instead compared to one another. Samples are ranked from highest to lowest geometric mean viral load. For TAC‐B: library preparation failed for sample HBV13; samples HBV4, HBV9, and HBV19 had reads of poor quality for the original pipeline to process, so the mean base quality was dropped below 1.0. #: For PCR‐1, a negative water control was tested instead of the negative plasma control. Alphabetical superscripts (a, b, c) represent identical sequences. Numerical superscripts next to method identifiers indicate where the same laboratory performed the protocols.

All four PCR‐based protocols detected false‐positive HBV sequences in the negative control (genome coverage, 48%–100%); these were identified as different genotypes indicative of sample or assay contamination (Table S3). These protocols were performed across three different laboratories, with extraction undertaken at the coordinating centre for three of the four protocols. Notably, contaminating sequences were also detected in the lowest VL samples (< 50 IU/ml) for two protocols in which extraction was performed at the same centre and sequencing was performed in the same (separate) centre, with differing contaminant genotypes observed for the same samples across the two protocols. Some contaminants and false positives matched sequences of other ex vivo viruses (Table 1), but most were non‐identical. For PCR‐based methods other than PCR‐1, the proportion of contaminants or the lack of detection at the lowest VLs deterred the ability to ascertain true‐positive detection confidently. TAC methods had very few contaminating sequences, and no contaminating sequences were assembled from MTG methods.

Out of 23 samples, true‐positive HBV sequences of > 5% genome coverage were detected in 5–6 samples using MTG, 10–17 samples using TAC, 9–13 samples using PCR‐Illumina, and 16–19 samples using PCR‐ONT protocols, with ranges reflecting variation across protocols. TAC‐B detected HBV DNA in 13 samples; under the same pre‐capture conditions, MTG‐B detected 7 of these 13 samples, with lower coverage and read numbers, including the six highest‐coverage samples detected by TAC‐B.

### 3.2 HBV Base Counts and Genome Coverage

**Figure 1:**
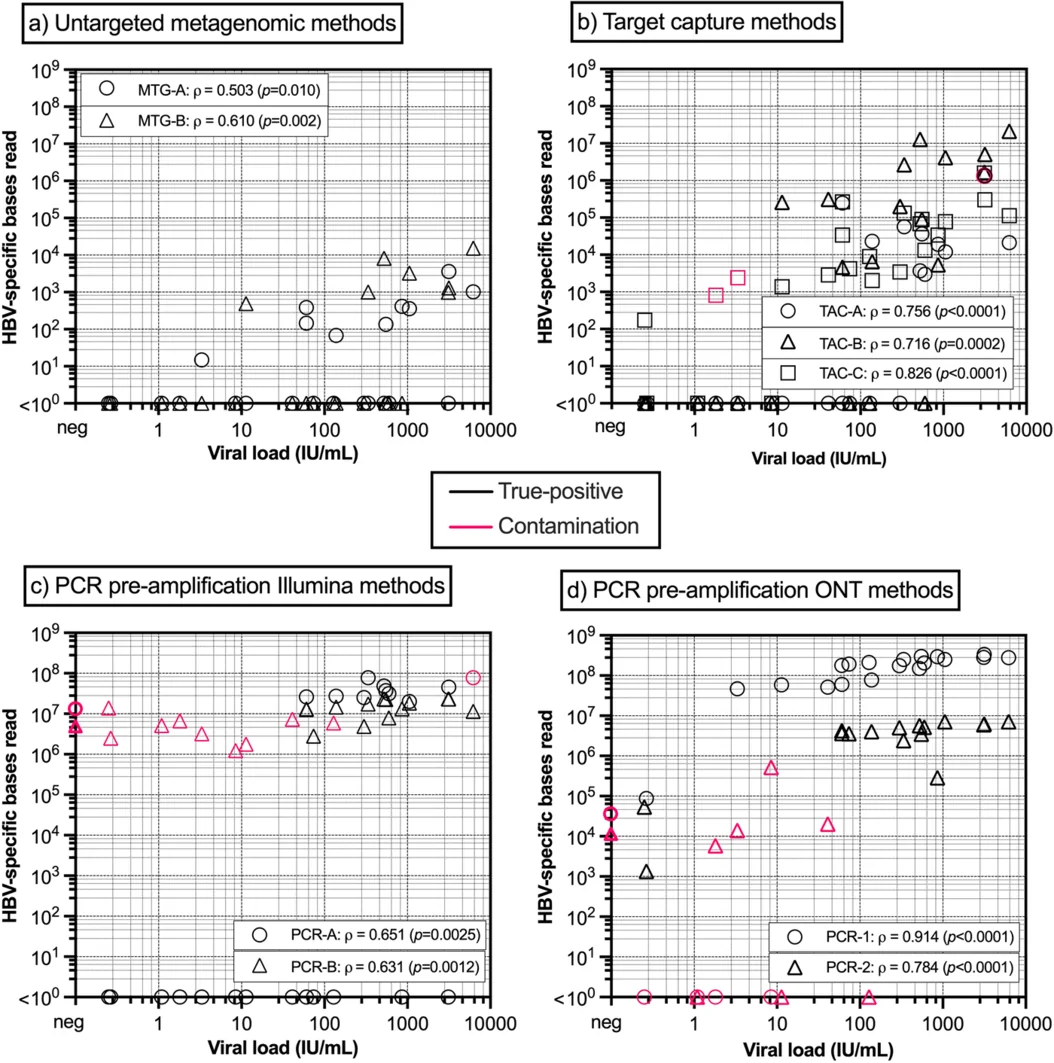
Relationship between viral loads and HBV‐specific bases read for each group of methods on a common log scale for (a) untargeted metagenomic methods, (b) target capture methods, (c) PCR pre‐amplification Illumina methods, and (d) PCR pre‐amplification ONT methods. Pink indicates contaminating sequences, including where HBV sequences were obtained for the negative HBV10 sample on the y‐axis. Samples not detected are found on the x‐axis. Spearman's correlation test with Benjamini‐Hochberg adjustment deduced the significance of the association between linear viral loads and base counts (ρ value and *p*‐values listed in boxes). HBV, hepatitis B virus; MTG, untargeted metagenomics; TAC, target capture; ONT, Oxford Nanopore Technologies.

The highest number of HBV‐specific bases was read in PCR methods (up to 10^9^), followed by TAC (up to 10^7^) and the lowest in MTG (up to 10^4^). A strong correlation was observed between base counts and VL using the PCR‐ONT and TAC methods, whereas this correlation was moderate when using the PCR‐Illumina and MTG methods (Figure 1). Base counts were similar between samples for the PCR‐Illumina methods. For PCR‐ONT, bases obtained for true‐positive samples with VL < 50 IU/mL ranged from 1 × 10^3^ to 5 × 10^7^. For TAC and MTG methods, base counts were much lower for samples with lower VL.

Empirical thresholds from observed relationships with VL, total HBV‐specific bases, and genome coverage were explored post‐hoc to aid discrimination between true‐positive signal and background contamination. These thresholds were specific to this dataset rather than representing analytical limits of detection. In this dataset, empirical thresholds for reliable identification for true‐positive sequences in the presence of background contamination were > 50 IU/mL or > 10^6^ bases for PCR‐ONT methods (except PCR‐1, for which no threshold could be defined), > 50 IU/mL or > 10^4^ bases for TAC methods, > 100 IU/mL or > 10^7^ bases for PCR‐Illumina methods, and > 3000 IU/mL or > 10^3^ bases for MTG methods.

**Figure 2:**
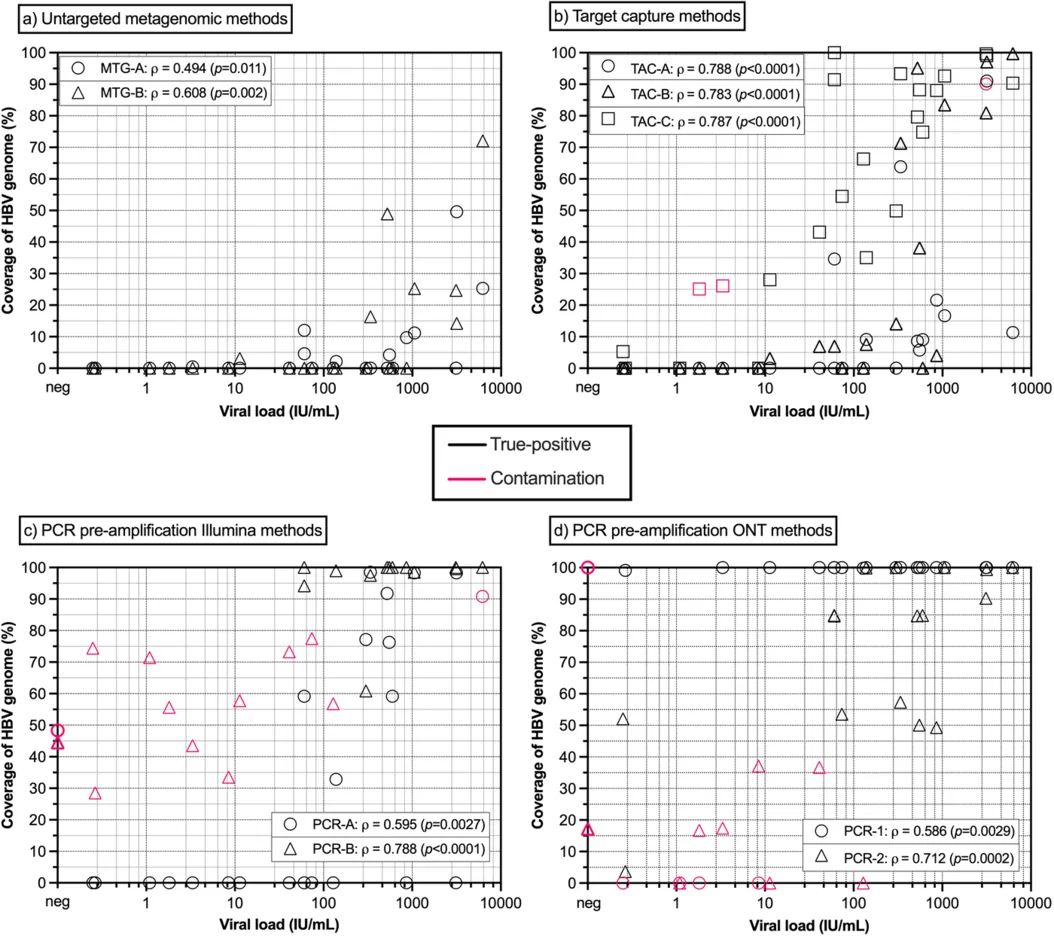
Relationship between viral loads and breadth coverage of HBV assembled sequences for each group of methods on a common axes scale for (a) untargeted metagenomic methods, (b) target capture methods, (c) PCR pre‐amplification Illumina methods, and (d) PCR pre‐amplification ONT methods. Pink indicates contaminating sequences, including where HBV sequences were obtained for the negative HBV10 sample on the y‐axis. Samples not detected are found on the x‐axis. Spearman's correlation test with Benjamini‐Hochberg adjustment deduced the significance of the association between linear viral loads and genome coverage (ρ value and *p*‐values listed in boxes). HBV, hepatitis B virus; MTG, untargeted metagenomics; TAC, target capture; ONT, Oxford Nanopore Technologies.

Complete or near‐complete genome sequences were assembled for all samples with VL > 10 IU/mL for PCR‐1 protocol, > 200 IU/mL for PCR‐B, > 1000 IU/mL for TAC‐C, and in none of the samples for MTG methods (Figure 2). In this dataset, threshold‐based approaches could be applied to distinguish true signal from contamination for some protocols, with approximate genome coverage thresholds of 40% for PCR‐2, 80% for PCR‐B, and 30% for TAC‐C; such thresholds were however not applicable to the two other protocols with contaminating sequences (PCR‐1 and PCR‐A).

**Figure 3:**
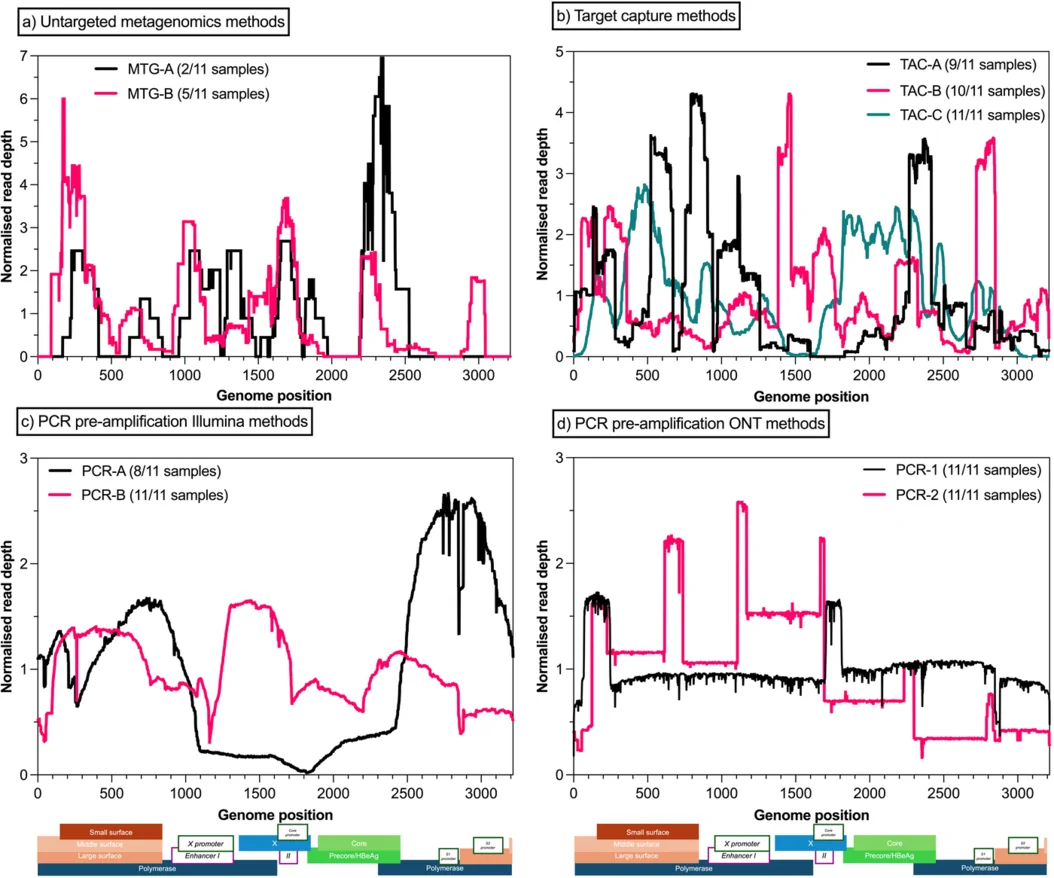
Normalised mean read depth of the HBV genome for each of the sequencing methods: (a) untargeted metagenomic methods, (b) target capture methods, c) PCR pre‐amplification Illumina methods, and d) PCR pre‐amplification ONT methods. For each sample, read depth at each genome position was calculated as the proportion of total HBV‐mapped bases detected at that position and multiplied by the total number of genome positions, such as the mean normalised depth across the genome was 1. For each protocol, the normalised read depth at each genome position was then averaged across included samples. Thus, y‐axis values below 1 represent positions with lower‐than‐average coverage, whereas values above 1 represent positions with higher‐than‐average coverage. The 11 highest viral load samples were included when detected, and only true‐positive sequences with more than 1000 total HBV‐specific bases read were included; the number of samples from each protocol that were included is indicated in each plot's key. Genome positions were based on the D00330 reference sequence. A genome diagram of HBV, drawn to the x‐axis scale, shows gene positions in shaded boxes and regulatory regions in unshaded boxes. MTG, untargeted metagenomics; TAC, target capture.

Inspection of the mean genome coverage revealed that the methods yielded uneven coverage across the genome (Figure 3). As no uniform coverage profile is expected a priori due to methodological and biological factors, comparisons were made on a relative basis across methods. PCR‐1 produced the most uniform genome coverage. Other PCR‐based methods had more regions of reduced coverage, including reduced amplification spanning the region from the start of the polymerase to the surface genes for PCR‐2, genome positions 1100 to 2100 for PCR‐A and the start of the surface gene for PCR‐B. Probe capture efficiency was highly variable across protocols, each showing areas of markedly low capture efficiency. Coverage of the HBV genome was even sparser for MTG methods. When analysing individual samples, there was considerable heterogeneity in the distribution of HBV genome coverage between protocols, without clear patterns (Figure S2). Although no obvious genotype‐related pattern was apparent, formal evaluation of genotype‐specific effects was limited by the small number of samples per genotype and by the fact that, among the 23 low VL samples, only a subset yielded sufficient HBV detection and genome coverage for meaningful comparison.

### 3.3 Heterogeneity

**Figure 4:**
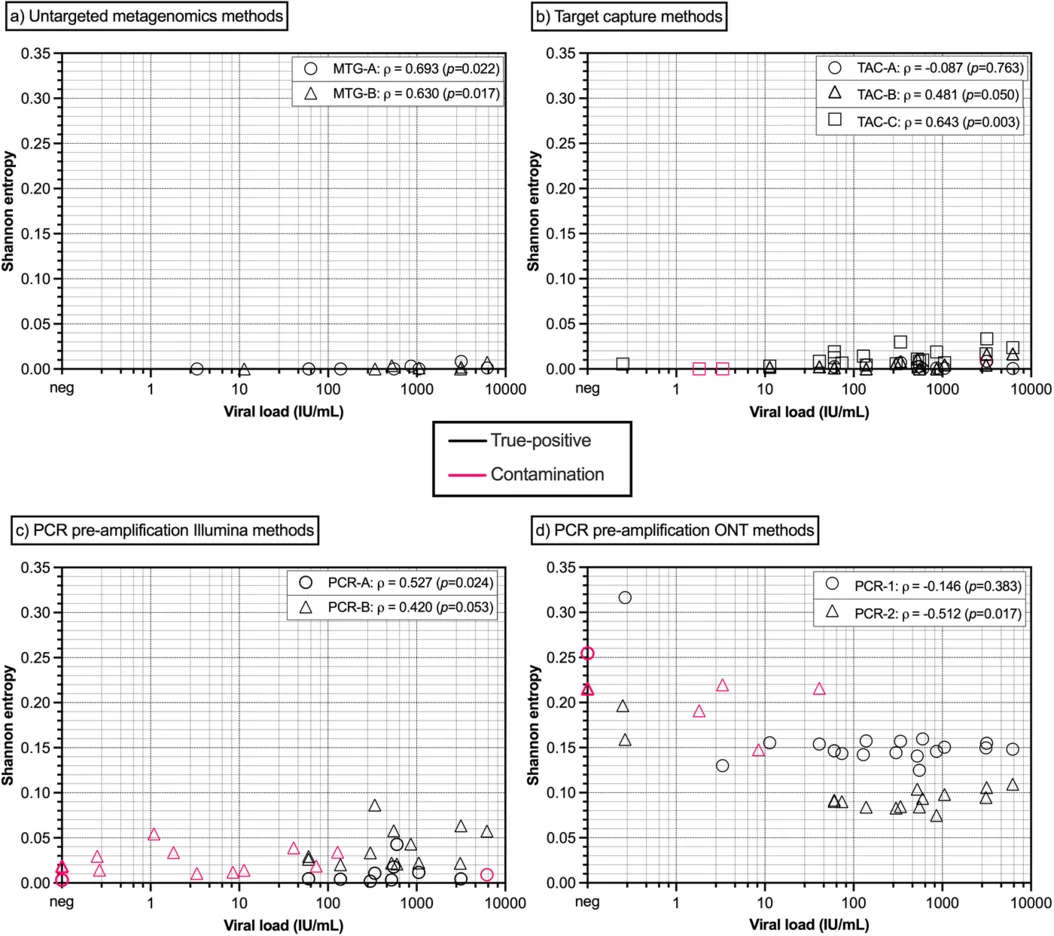
Relationship between viral loads and mean Shannon entropy values for polymorphic sites for each group of methods on a common axes scale for a) untargeted metagenomic methods; b) target capture methods; c) PCR pre‐amplification Illumina methods; and d) PCR pre‐amplification ONT methods. Pink indicates contaminating sequences, including where HBV sequences were obtained for the negative HBV10 sample on the y‐axis. Samples not detected are found on the x‐axis. Spearman's correlation test with Benjamini‐Hochberg adjustment deduced the significance of the association between linear viral loads and entropy values (ρ value and *p*‐values listed in boxes). HBV: hepatitis B virus; MTG: untargeted metagenomics; ONT, Oxford Nanopore Technologies; TAC, target capture.

The diversity of base counts at each site was quantified through calculations of Shannon entropy. As no expected distribution of intra‐host diversity could be assumed, Shannon entropy was used for comparison of read heterogeneity between methods. Variability in Shannon values was observed between the NGS methods for the same samples, which may reflect variations in the quantities of base counts by each method. MTG methods showed the least intra‐sample HBV sequence heterogeneity, with a maximum mean Shannon entropy of 0.01, followed by the TAC methods (up to 0.04) and the PCR‐Illumina methods (up to 0.09). Heterogeneity was extremely high (up to 0.32) for PCR‐ONT methods (Figure 4). However, the presence of ambiguous bases in sequences from PCR‐ONT methods did not affect the accuracy of consensus sequences generated from the reads. Diversity was higher when VL was greater for PCR‐Illumina methods, but was lower when VL was higher for PCR‐ONT methods.

**Figure 5:**
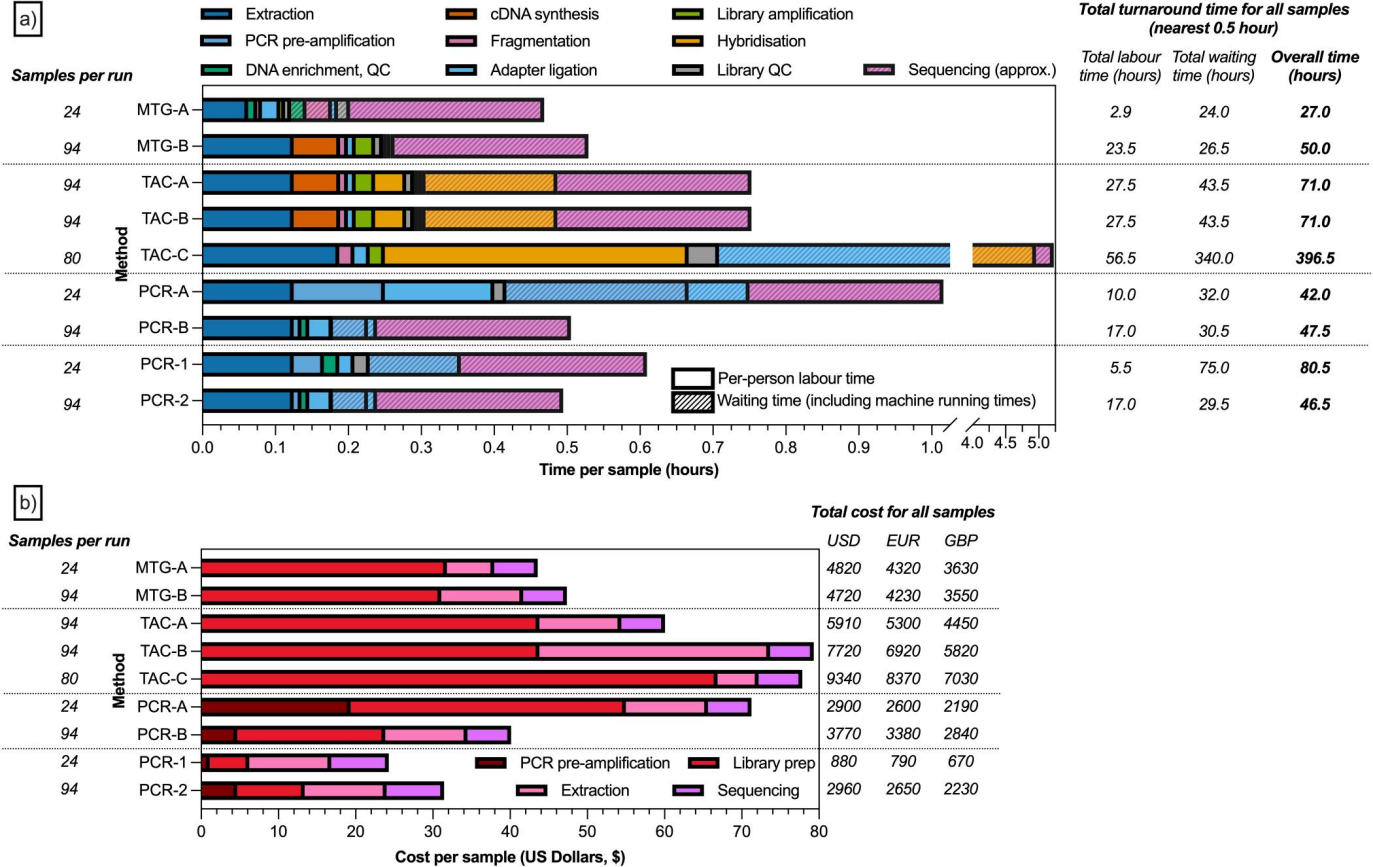
Comparison of (a) time taken and (b) cost for each protocol based on the total time and costs for a protocol‐specific representative number of samples included in a typical run. For Figure 5a, coloured bars to the left of the plot show per‐person labour time per sample, whereas the same‐coloured bars with lighter‐coloured diagonal lines to the right show the waiting time per sample, which may include machine running times. For visual comparison, sequencing time and costs per sample were standardised to the shortest and cheapest Illumina or Nanopore protocol; the overall times reported include the full sequencing durations as recorded by each laboratory. For clinical purposes, PCR‐1 is normally run overnight on the MinIon flow cell (average of 16 h), but was run for 72 h for this study. Costs were standardised using a 1:0.8959:0.7531 USD:EUR:GBP exchange rate, as of May 18, 2025, and total costs were rounded to the nearest 10. EUR, Euro; GBP, Great British Pound; MTG, untargeted metagenomics; QC, quality control; TAC, target capture; USD, United States Dollar.

### 3.4 Co‐Detection

Protocol‐specific bioinformatic pipelines were employed to determine the presence, read counts, and genome coverage of other viruses for the TAC and MTG methods, which simultaneously screened for multiple viruses from generated sequences (Table 2). Altogether, anellovirus sequences were detected in 22/24 samples by the five NGS protocols. Confirmatory PCR for alpha, beta, and gammatorqueviruses revealed the presence of DNA sequences of all three anellovirus genera in all 24 samples (20/24 αTTV‐positive αTTV; 22/24 βTTV‐positive; 17/24 γTTV‐positive), all with relatively high VLs (Ct values ranged from 4.7 to 26.1). Sanger sequencing of amplicons generated by nested PCR from randomly selected anellovirus‐positive samples confirmed the presence of different strains from each genus.

**Table 2:** Detection of other viruses by the targeted capture and untargeted metagenomics methods, showing deduplicated read counts (genome coverage %).

Sample	Virus	MTG‐A	MTG‐B^1^	TAC‐A^1^	TAC‐B^1^	TAC‐C	PCR
HBV1	AV	43 (54%)	—	6 (14%)	3 (3%)	37 (22%)	Ct 13.1 (αTTV)
	HHV7	—	—	—	—	885 (10%)	Negative
HBV2	MCPyV	—	—	—	—	14 (20%)	Negative
	AV	—	—	—	—	35 (21%)	Ct 9.6 (αTTV), Ct 8.0 (βTTV), Ct 21.1 (γTTV)
	HHV7	—	—	—	—	649 (10%)	Negative
HBV3	AV	9 (20%)	2 (1%)	7 (20%)	19 (12%)	167 (49%)	Ct 6.3 (αTTV), Ct 5.8 (βTTV), Ct 8.5 (γTTV)
	EBV	—	—	—	—	321 (6%)	Negative
	HHV7	—	—	—	—	1699 (9%)	Negative
HBV4	AV	26 (49%)	—	11 (20%)	14 (16%)	117 (45%)	Ct 10.9 (αTTV), Ct 4.7 (βTTV), Ct 13.0 (γTTV)
	Simian adenovirus 25	7 (0.74%)	—	—	—	—	=
HBV5	AV	—	5 (8%)	—	20 (16%)	15 (15%)	Ct 22.2 (αTTV), Ct 11.0 (βTTV)
	JCPyV	—	—	—	—	63 (52%)	=
HBV6	AV	6 (21%)	44 (40%)	7 (12%)	310 (75%)	84 (36%)	Ct 7.2 (βTTV)
	MCPyV	—	—	—	—	126 (65%)	152 copies/mL
	HPyV6	—	—	—	4 (4%)	26 (30%)	Negative
	HHV7	—	—	—	—	565 (10%)	Negative
	JCPyV	—	—	—	—	70 (56%)	=
HBV7	AV	—	2 (2%)	13 (6%)	11 (24%)	14 (17%)	Ct 7.2 (γTTV)
	HPV‐2	—	—	—	—	10 (8%)	**=**
	HHV7	—	—	—	—	1112 (9%)	Negative
	JCPyV	—	—	—	—	34 (41%)	=
HBV8	EBV	12 (0.2%)	—	42 (14%)	3 (2%)	2020 (39%)	Ct 35.9
	AV	—	1 (1%)	1 (5%)	—	—	Ct 12.5 (αTTV), Ct 8.3 (βTTV), Ct 21.4 (γTTV)
	MCPyV	—	—	—	—	35 (31%)	Negative
HBV9	AV	—	—	3 (4%)	1 (1%)	—	Ct 15.0 (αTTV), Ct 8.9 (βTTV), Ct 19.2 (γTTV)
	HHV6	—	—	—	1 (1%)	1626 (15%)	Ct 38.2
HBV10	AV	—	3 (1%)	3 (4%)	1 (9%)	—	Ct 12.6 (αTTV), Ct 10.7 (βTTV)
	HHV7	—	—	—	—	730 (7%)	Negative
HBV11	AV	—	—	1 (6%)	2 (13%)	22 (10%)	Ct 6.1 (αTTV), Ct 5.9 (βTTV), Ct 22.5 (γTTV)
	EBV	—	—	—	—	774 (7%)	Negative
	HHV7	—	—	—	—	805 (7%)	Negative
HBV12	KSHV	18 (0.63%)	—	1 (5%)	—	7353 (93%)	Ct 36.6
	EBV	—	—	—	1 (1%)	—	Negative
	HHV7	—	—	—	—	1324 (9%)	Negative
	AV	—	2 (1%)	—	—	—	Ct 26.1 (αTTV), Ct 13.3 (βTTV)
HBV13	MCPyV	—	—	—	—	16 (15%)	Negative
	TTV	—	3 (2%)	—	—	20 (24%)	Ct 11.9 (αTTV), Ct 5.1 (βTTV), Ct 13.0 (γTTV)
	HHV7	—	—	—	—	825 (11%)	Negative
HBV14	AV	—	8 (11%)	—	88 (33%)	—	Ct 7.0 (αTTV), Ct 10.1 (βTTV)
	JCPyV	—	—	—	—	10 (10%)	=
HBV15	JCPyV	—	—	—	—	69 (59%)	=
	AV	7 (9.6%)	7 (1%)	—	16 (35%)	11 (8%)	Ct 12.4 (αTTV), Ct 11.4 (βTTV), Ct 15.2 (γTTV)
	EBV	—	—	—	1 (1%)	—	Negative
HBV16	AV	—	1 (1%)	—	1 (12%)	—	Ct 13.8 (αTTV), Ct 9.1 (βTTV)
HBV17	HHV7	—	—	—	—	413 (9%)	Negative
	AV	—	—	—	—	—	Ct 13.3 (βTTV), Ct 15.3 (γTTV)
HBV18	AV	—	—	1 (3%)	1 (1%)	—	Ct 12.9 (αTTV), Ct 11.4 (βTTV), Ct 21.0 (γTTV)
HBV19	AV	—	—	—	—	—	Ct 19.1 (αTTV), Ct 16.2 (βTTV), Ct 9.5 (γTTV)
HBV20	AV	27 (41%)	—	8 (13%)	2 (8%)	25 (18%)	Ct 12.2 (αTTV), Ct 11.5 (βTTV), Ct 6.9 (γTTV)
HBV21	TTV	—	—	1 (4%)	3 (9%)	—	Ct 11.9 (βTTV), Ct 9.4 (γTTV)
	HPV	—	—	—	—	17 (15%)	=
	HHV7	—	—	—	—	833 (9%)	Negative
	MCPyV	—	—	—	—	—	219 copies/mL
HBV22	AV	—	—	—	8 (11%)	—	Ct 11.4 (αTTV), Ct 12.1 (βTTV), Ct 21.4 (γTTV)
	HHV6	—	—	—	—	1070 (7%)	Negative
HBV23	AV	—	2 (1%)	11 (35%)	22 (26%)	56 (30%)	Ct 5.8 (αTTV), Ct 6.4 (βTTV), Ct 19.8 (γTTV)
	EBV	—	—	—	—	212 (6%)	Negative
	HHV6	—	—	—	—	1535 (7%)	Negative
	HHV7	—	—	—	—	761 (5%)	Negative
	MCPyV	—	—	—	—	10 (8%)	Negative
HBV24	AV	—	2 (1%)	22 (28%)	4 (6%)	31 (21%)	Ct 7.8 (αTTV), Ct 7.0 (βTTV), Ct 13.5 (γTTV)
	HHV6	—	—	—	—	616 (11%)	Negative
	HPyV7	—	—	—	—	13 (12%)	=
	HPyV6	—	—	—	—	27 (24%)	Negative
	KSHV	—	—	—	—	226 (5%)	Negative
	HHV7	—	—	—	—	326 (5%)	Negative

*Note:* Results reported for TAC‐C are for viruses where at least 10 reads were obtained. More than one TTV species may have been identified for MTG‐A, but the most abundant was included. The furthest right column shows the PCR results, where PCR was performed for all samples when two or more methods detected a virus in the same sample. These PCRs were specific for alpha, beta and gamma‐torquevirus, Merkel cell polyomavirus, and herpesviruses. ‘‐’ denotes where the method did not detect the virus, ‘=’ denotes where the PCR was not performed. A numerical superscript indicates where the same laboratory performed the protocols.

Abbreviations: AV, anellovirus; EBV, Epstein‐Barr virus; HHV, human herpesvirus; JCPyV, John Cunningham virus; KSHV, Kaposi's sarcoma‐associated herpesvirus; MCPyV, Merkel cell polyomavirus; TTV, torquetenovirus.

Various human herpesviruses were detected in 17/24 samples across the protocols at < 50% genome coverage, and were detected by more than one protocol for three samples (EBV in 4/5 protocols in two laboratories for sample HBV8; HHV6 in 2/5 protocols in two laboratories for HBV9; KSHV in 3/5 protocols in three laboratories for HBV12). Real‐time PCR confirmed the presence of these herpesviruses in these three samples, but no other sample was HHV PCR‐positive, including the 13 samples where TAC‐C reported HHV‐7 DNA sequences. Polyomavirus DNA sequences were detected in 11/24 samples using NGS protocols; however, they were only detected by more than one protocol in one sample (HPyV6, detected by two protocols in two laboratories in HBV6). HyPV6 was, however, not detected by real‐time PCR. Overall, TAC methods detected a greater number of viruses at higher read counts and coverage than MTG methods.

### 3.5 Time and Costs

The per‐person labour and waiting times varied between protocols (Figure 4a). The turnaround time was shortest for MTG and most PCR‐based protocols. TAC methods took longer, mostly due to overnight hybridisation steps. Costs were lowest for PCR‐ONT methods, higher for PCR‐Illumina methods, followed by MTG methods, whilst TAC methods had the greatest cost (Figure 4b).

## 4 Discussion

Findings from our multicentre study provide an overview of currently available NGS methodologies for sequencing low VL HBV clinical samples. They highlight the HBV‐specific sensitivities and potential applicability in clinical practice.

While all evaluated methods demonstrated varying capacities to generate HBV sequences, protocols incorporating pre‐amplification steps showed greater sensitivity at low VLs. Reliable detection of HBV at VL ~ 10 IU/mL (for PCR‐1 and TAC‐C) or ~100 IU/mL (for other pre‐amplification protocols) would be sufficient for most HBV infections encountered in clinical settings. For detecting and quantifying samples with very low VL, such as those associated with occult HBV [15], conventional real‐time or nested PCR remained essential. Previous investigations of PCR‐1 and PCR‐2 also showed successful amplification of full HBV genomes at VL < 30 IU/mL [6, 13], and alternative probe‐capture methods observed a similar 50 copies/ml limit of detection for HBV [23] and 90%–100% coverage for a range of DNA and RNA viruses at ≥ 1000 copies/mL [24, 25]. The substantially reduced depth of coverage for MTG methods we observed has been well‐described for a range of viruses [10, 11, 12]. However, the method retains its value in other applications due to its ability to detect unanticipated or potentially entirely novel pathogens without a requirement to pre‐select targets in capture and PCR‐assisted NGS methods [2].

Although detection sensitivity was similar, genomes generated from PCR‐based methods were near‐complete compared to lower genome coverage from TAC methods for our low VL samples. As an inexpensive method for sequencing whole HBV genomes, PCR‐ONT NGS may be a beneficial screening tool for the HBV and microbiology fields, especially in low‐ and middle‐income countries [26], when ONT allows preliminary analysis before a run is completed: highly advantageous in terms of turnaround time for high VL samples. Our findings contrast with a similar study on hepatitis C virus, which concluded that PCR‐based NGS was relatively laborious [12]; improvements in technologies and workflows could explain the differences found. However, PCR‐based NGS would be more challenging for more divergent and/or larger viruses than HBV, as primer design for whole‐genome amplification can be difficult, and amplicon drop‐out necessitating primer re‐design is not uncommon. Variables that may have affected the methods' detection capabilities in this study include extraction from larger volumes of plasma (as previously found with PCR‐1 [13] and TAC‐A/B protocols [22]), having greater sample representation and fewer samples for library preparation, and sequencing more gigabases per sample. For PCR‐based methods, sensitivity is affected less by the sequencing platform and more by the effectiveness of the PCR for genome pre‐amplification, particularly the conservation of primer‐binding sites. A nested PCR strategy with shorter amplicons would improve sensitivity if WGS is not required. For TAC methods, sensitivity may have been affected by factors such as probe design, hybridisation, and wash temperatures. As with the detection of viruses around the limit of detection of conventional PCR assays, the stochastic effect around the limits of detection of NGS protocols affects the detection of samples with low VL (consistent with the marked variability in genome detection and coverage observed between protocols), albeit at a higher detection limit than conventional PCR.

Although the sensitivity of PCR‐based NGS methods is sufficient for most clinical cases of HBV, the finding of low‐level HBV DNA contamination compromised their diagnostic reliability at the lowest VLs. Without applying a minimum threshold of 10 for consensus base calling to account for potential background contamination or sequencing errors, HBV sequences would have been detected in all samples (24/24) using PCR‐based methods; however, contaminant HBV sequences would also have appeared more frequently (Table S4). The detection of contaminating sequences has been reported in other studies comparing NGS protocols [11, 24], highlighting contamination as a persistent obstacle to the reliable characterisation of low VL genomes. Notably, one planned protocol could not be included in this study because the background level of contamination exceeded the VLs of the sample panel.

Investigating the exact sources and mechanisms of contamination was beyond the scope of this study. The negative control was confirmed to be HBV‐negative prior to processing. Given the study's primary aim of comparing sequencing methodologies and the limited availability of plasma samples, additional negative plasma controls were not included in this study, limiting our ability to comprehensively assess protocol‐ or laboratory‐specific contamination in each laboratory. Contamination was likely attributable to protocol‐ or laboratory‐related factors rather than an inherent limitation of NGS itself; low VLs and complex sequencing workflows primarily increase the detectability of such contamination rather than its occurrence. This interpretation is supported by the detection of identical sequences across multiple samples processed within the same protocol or laboratory (Table 1). The variation observed in both the number and characteristics of contaminant strains among protocols indicates that contamination events in low VL samples are relatively frequent but highly context‐dependent. In the presence of background contamination, stricter analytical thresholds can reduce sensitivity but improve the reliability of positive calls. Such context‐ and protocol‐specific measures may include increasing the minimum read depth required for consensus base calls, enforcing minimum genome coverage or VL thresholds, and setting defined read count criteria relative to negative controls (Table S5). Importantly, minimising contamination in complex NGS workflows requires extensive experience and rigorous laboratory practice [27], such as using separate PCR workspaces and tubes, exercising meticulous pipetting, and including appropriate negative plasma controls. Indeed, very high VLs that may be encountered in HBV‐positive specimens, particularly those that are HBeAg‐positive, together with the stability and persistence of HBV DNA in the laboratory environment, may increase the risk of inadvertent cross‐contamination unless stringent handling procedures are maintained from the outset.

Intra‐host viral heterogeneity and the detection of minority variants are important considerations, particularly for identifying emerging drug resistance mutations, diagnostic escape mutations, or other adaptive changes. Low VL may arise at different stages of HBV infection, which can be associated with differing levels of intra‐host viral diversity. The anti‐HBe positive profile of the samples suggests infection in the low‐replication phase potentially associated with increased diversity [28]. However, Shannon entropy that quantified population diversity varied substantially between methods, and its relationship with VL differed by approach. PCR‐based methods generated more HBV bases read and therefore provided a greater opportunity to detect low‐frequency variants, including both true within‐sample variants and artefactual changes introduced by transcription errors during amplification or sequencing. For PCR‐Illumina, the increase in entropy with higher VL may therefore reflect improved detection of genuine intra‐host sequence diversity. By contrast, the very high entropy values observed for PCR‐ONT are difficult to interpret biologically, as the error rates among ONT base‐calling algorithms used in this study (during 2023–2024) likely obscured the true relationship between VL and biological heterogeneity, particularly in lower VL samples where such errors would contribute a greater proportion of the observed variation. Although ONT sequencing may ultimately prove valuable for assessing within‐sample diversity because long reads can identify linked variation within near‐complete viral genomes, accurate identification of minority variants remained challenging in this study. These errors are unlikely to impact interpretation for most clinical and public health purposes, but reliable variant calling from mapped ONT reads remains challenging and requires careful bioinformatics processing to distinguish artefactual substitutions from true minority variants [29]. Subsequent improvements in R10 flow cells and base‐calling algorithms, after our experiments were performed, are likely to enhance sequencing accuracy and reduce base count variability [30, 31].

NGS methods involving PCR amplification are inherently limited to the characterisation of one known target pathogen. Their use for whole genome characterisation is compromised for viruses with long genomes such as herpesviruses, where multiple, overlapping PCRs may be required, or for targets which are highly genetically heterogeneous and which may lack conserved sites for primer binding across the genome, such as anelloviruses. Metagenomic methods possess clear advantages in their breadth of detection, with frequent co‐detections of multiple anelloviruses, human herpesviruses, and polyomaviruses in the samples. The 100% (24/24) prevalence of anellovirus DNA detection by PCR was consistent with previous studies, which have shown that most immunocompetent individuals carry the virus in their blood and at high VLs in patients with HBV infection [32]. The low detection rate by all TAC protocols may have originated from the extreme genetic heterogeneity of the anellovirus species and genera that were not adequately represented among the probes used for target capture [33]. Our detection rates of polyomaviruses and HHVs were comparable to those reported in population studies [34, 35]. The high detection rate of HHVs and polyomaviruses with TAC‐C compared to fragmented PCR could potentially be explained by the presence of incomplete or highly fragmented genome sequences derived from degraded viral DNA from disintegrating persistently infected cells [36].

Our study focused on HBV samples with low VLs; including samples with higher VLs would likely have increased the proportion achieving high base counts and complete genome coverage, while reducing the relative impact of contamination on detection reliability. Additionally, only one sample each from HBV genotypes B and C was included. Although the sample panel reflected the genotype distribution in Europe, the sample set used for assessment of protocol performance did not reproduce the global diversity of HBV and may reduce the generalisability of these findings to settings where genotypes B and C are more prevalent. DNA extraction was not standardised across participating laboratories, as laboratories either used their routine extraction workflows or, where requested, received extracted nucleic acids instead of plasma. Whilst this introduced an additional source of pre‐analytical variability [15, 37], the study result reflects current clinical practice, where laboratories use different extraction methods that may be validated locally but not harmonised across centres. Although we centralised some of the bioinformatics to facilitate comparison, pipeline‐specific steps performed by each laboratory could have influenced performance, including the choice of taxonomic classifier [10]. In a follow‐up study, we plan to evaluate the performance of different bioinformatic pipelines on the raw data generated in the current study to ascertain optimal tools for HBV characterisation.

In conclusion, this study provides an overview of the HBV‐specific performance of currently used NGS methodologies and their potential applicability for sequencing low VL clinical samples. NGS approaches incorporating PCR amplification demonstrate sufficient sensitivity for most HBV infections typically encountered in clinical practice, where VLs exceed approximately 10 IU/mL. However, the presence of low‐level contamination remains a challenge in complex sequencing workflows, restricting reliable genome identification at the lowest VLs where contaminating reads have a proportionally greater impact. Future efforts should focus on identifying and mitigating sources of background contamination and a protocol design that prevents DNA carryover from high VL samples. These will enable broader clinical implementation of NGS as a valuable tool for characterising HBV and other viral pathogens in diverse diagnostic settings.

## Author Contributions

Conceptualisation: Heli Harvala, Peter Simmonds, Michael X. Fu. Formal analysis: Michael X. Fu, Heli Harvala, Peter Simmonds, William L. Irving. Investigation: Michael X. Fu, Maria F. Perdomo, Sheila F. Lumley, Johan Ringlander, Kai Kean, Kaitlin Reid, Richard Mayne, Oscar Enrique Torres Montaguth, Leysa Forrest, Sarah Buddle, Johannes C. Bothao, Johannes C. BothaS, Zachery Dickson, Chris Kent, Haiting Chai, Matthew Byott, Leo Hannolainen, Shannah Secret, George Airey, Klaus Hedman, Monique I. AnderssonA, Eleni Nastouli, Johannes C. Bothar, Philippa C. Matthews, Tanya Golubchik. Writing – Original Draft: Michael X. Fu. Writing – Review and Editing: all authors. Supervision: Heli Harvala, Peter Simmonds, Monique I. Andersson. All authors approved the final version of the submitted manuscript.

## Conflicts of Interest

The authors declare no conflicts of interest.

## Supporting information


Supporting File

## Data Availability

The data supporting the findings of this study are included in the manuscript. Raw sequencing data are openly available through the NCBI BioProject accession number PRJNA1364201, including BioSample accession numbers SRR36116996 to SRR36117201. Consensus full‐length genome sequences have been deposited in GenBank under accession numbers PV786119 to PV786132.
